# Impact of Reduced Maternal Exposures to Wood Smoke from an Introduced Chimney Stove on Newborn Birth Weight in Rural Guatemala

**DOI:** 10.1289/ehp.1002928

**Published:** 2011-06-07

**Authors:** Lisa M. Thompson, Nigel Bruce, Brenda Eskenazi, Anaite Diaz, Daniel Pope, Kirk R. Smith

**Affiliations:** 1 Family Health Care Nursing, School of Nursing, University of California, San Francisco, California, USA; 2 Environmental Health Sciences, School of Public Health, University of California, Berkeley, California, USA; 3 Department of Public Health and Policy, Institute of Psychology, Health and Society, University of Liverpool, Liverpool, United Kingdom; 4 Center for Environmental Research and Children’s Health, School of Public Health, University of California, Berkeley, California, USA; 5 Centro de Estudios en Salud, Universidad del Valle, Guatemala City, Guatemala

**Keywords:** carbon monoxide, household air pollution, low birth weight, maternal malnutrition, RESPIRE trial, seasonality

## Abstract

Background: A growing body of evidence indicates a relationship between household indoor air pollution from cooking fires and adverse neonatal outcomes, such as low birth weight (LBW), in resource-poor countries.

Objective: We examined the effect of reduced wood smoke exposure in pregnancy on LBW of Guatemalan infants in RESPIRE (Randomized Exposure Study of Pollution Indoors and Respiratory Effects).

Methods: Pregnant women (*n* = 266) either received a chimney stove (intervention) or continued to cook over an open fire (control). Between October 2002 and December 2004 we weighed 174 eligible infants (69 to mothers who used a chimney stove and 105 to mothers who used an open fire during pregnancy) within 48 hr of birth. Multivariate linear regression and adjusted odds ratios (ORs) were used to estimate differences in birth weight and LBW (< 2,500 g) associated with chimney-stove versus open-fire use during pregnancy.

Results: Pregnant women using chimney stoves had a 39% reduction in mean exposure to carbon monoxide compared with those using open fires. LBW prevalence was high at 22.4%. On average, infants born to mothers who used a stove weighed 89 g more [95% confidence interval (CI), –27 to 204 g] than infants whose mothers used open fires after adjusting for maternal height, diastolic blood pressure, gravidity, and season of birth. The adjusted OR for LBW was 0.74 (95% CI, 0.33–1.66) among infants of stove users compared with open-fire users. Average birth weight was 296 g higher (95% CI, 109–482 g) in infants born during the cold season (after harvest) than in other infants; this unanticipated finding may reflect the role of maternal nutrition on birth weight in an impoverished region.

Conclusions: A chimney stove reduced wood smoke exposures and was associated with reduced LBW occurrence. Although not statistically significant, the estimated effect was consistent with previous studies.

Low-birth-weight (LBW; < 2,500 g) infants are at high risk for morbidity and mortality in infancy and childhood (Ashworth 1998; Lira et al. 1996; Luke et al. 1993) as well as chronic diseases in adulthood (Reyes and Manalich 2005; Thompson 2007). More than 95% of LBW infants are born in low-income countries (Lawn et al. 2010; United Nations Children’s Fund and World Health Organization 2004). In 2002, the World Health Organization (WHO) estimated that 12% of infants born in Guatemala were LBW [Pan American Health Organization (PAHO) 2007], although this is likely to be an underestimation of national rates, because those most likely to be LBW and least likely to be weighed at birth are rural, poor, indigenous children born at home.

Solid fuels, including coal, wood, crop residues, and animal dung, are used by about half the world’s population and by 90% of people residing in rural areas of low-income countries (Smith et al. 2004). Traditional cooking stoves are typically inefficient at combusting solid fuel and often lead to high exposures to particulate matter (PM) and carbon monoxide (CO). In Guatemala, where 83% of the rural households use wood for cooking fuel (Pruss-Ustun et al. 2008), 24-hr kitchen concentrations of PM_2.5_ (PM ≤ 2.5 μm in aerodynamic diameter) from wood fires can range from 100 μg/m^3^ in homes using chimney stoves in good condition to 1,000 μg/m^3^ in homes using open fires. These concentrations are orders of magnitude higher than those in homes where tobacco cigarettes are smoked, where daily mean concentrations of PM_2.5_ vary between 25 and 40 μg/m^3^ (Wallace 1996). Mean concentrations of CO over a 24-hr period are also high (ranging from 2 to 50 ppm in households using solid cooking fuels) and often exceed the health-based WHO guidelines of 9 ppm (in an 8-hr period) (Bruce et al. 2000; Saksena et al. 2003). Depending on source strength, ventilation, and other factors that mediate or magnify exposure, wood fires typically yield levels of exposure to PM and CO that are much higher than environmental (or secondhand) tobacco smoke (ETS). ETS has been linked to mean birth weight reductions of 33 g [95% confidence interval (CI), 16–51 g] and to a 22% increase in the prevalence of LBW infants (Leonardi-Bee et al. 2008).

Associations between maternal exposures to household air pollution (HAP) and LBW have been described in children in Guatemala (Boy et al. 2002), Zimbabwe (Mishra et al. 2004), Pakistan (Siddiqui et al. 2008), and India (Tielsch et al. 2009). In previous studies, all exposures to solid fuel smoke were estimated from self-report of fuel and stove type used. Thus, the level of exposures to specific household air pollutants is not known.

The purpose of this study was to determine the impact of reduced HAP from an introduced chimney stove on the prevalence of LBW infants in rural Guatemala. We assessed exposure with personal measures of CO during pregnancy. For this study, we conducted a post hoc analysis to examine the birth weight of Guatemalan infants whose mothers participated in the RESPIRE (Randomized Exposure Study of Pollution Indoors and Respiratory Effects) trial. The primary aim of RESPIRE was to examine the incidence of acute lower respiratory infection among children < 18 months of age in households randomized to receive a wood-fueled chimney-stove intervention (referred to locally as the *plancha*) compared with control families who continued to use traditional open fires (Smith et al. 2006).

## Materials and Methods

Between October 2002 and December 2004, the RESPIRE trial was conducted among 534 households (with 537 children) in 23 rural Guatemalan communities. Most participants (96%) were members of the Mam-Mayan linguistic group. Households were recruited if they used open wood fires for cooking and had either a pregnant women or a child < 4 months of age. Half of the study homes were randomly selected to receive the chimney stove (intervention group). The other half of the homes continued to use an open fire (control group) until their child reached 18 months, or until the study ended, at which time the family was offered a chimney stove.

**Figure 1:**
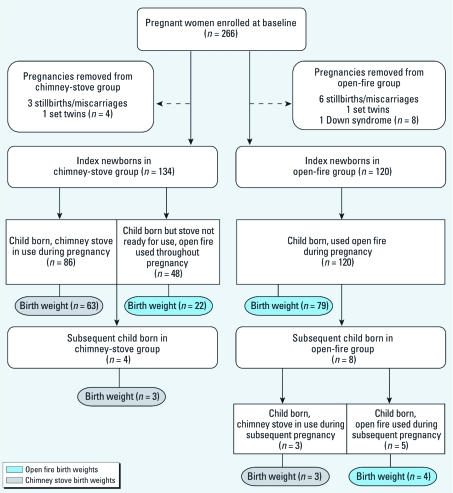
Study participants and total number of birth weights measured within 48 hr.

A total of 266 of the 534 women were enrolled during pregnancy, with the remaining children recruited at < 4 months of age. After enrollment, pregnant women received a baseline pregnancy examination by a physician employed by the study, which included a medical history, urine analysis, blood pressure measurement, and estimate of fetal growth (fundal height measurement). Fieldworkers visited women weekly in their homes, and women were referred to physicians if the women reported pregnancy complications. Among the enrolled pregnant women, 254 women delivered singleton, live births (five miscarriages, four stillbirths, two pregnancies with multiple gestations, and one child with Down syndrome were excluded). Twelve additional births occurred among these women during the study period. Figure 1 summarizes characteristics of pregnant women and births.

Among the 254 women, 240 participated in an ancillary study of adult women’s lung function. In this study, height and nonpregnant weight were measured (Diaz et al. 2007). Height was measured using a seca portable stadiometer (model 214; seca united kingdom, Birmingham, UK). Weight was measured using a Taylor Precision Tech Lithium electronic scale (model 7308; Taylor Precision Products, Las Cruses, NM, USA).

Once RESPIRE staff were notified of a birth, either by a fieldworker at the weekly home visit or by a family member who contacted project staff, the project manager (A.D.) attempted to visit the home to weigh the newborn within 48 hr. Sometimes practical issues made it difficult to reach the home to carry out measurement, such as delayed notification by family members, minimally skilled birth attendants who did not routinely weigh newborns, and communities located several hours away from the project office. Thus, of the 254 newborns, 208 (81.8%) birth weights were measured within 1 week, including 174 (68.5%) measured within the first 48 hr. Because newborns can lose up to 5–10% of their birth weight in the first week, we restricted our birth weight analysis to the 174 newborns measured within 48 hr of birth. Birth weight was measured in grams using a calibrated Siltec BS1 baby scale with 10-g readability (model 0309; Dogain Instruments, Inc., Santa Clara CA, USA). Infants wore a light shirt provided by project staff to standardize clothing weight. Gestational age was not assessed.

Intention-to-treat analysis assumes that those who were randomized to receive the intervention stove had lower exposure to HAP than did those in the control group who continued to use open fires. However, because the RESPIRE trial was designed to monitor infant respiratory illness, many of the pregnant women were recruited in the later stages of pregnancy. They received the stove, which required a 5-week drying period after construction, shortly before, or even a few days after, delivery. Therefore, birth weight outcome analyses were based on actual stove type in use during the pregnancy during the observation period and not based on randomization, resulting in 69 births to women who used a chimney stove during pregnancy and 105 births to women who used an open fire during pregnancy (174 births to 164 women).

Personal exposure to CO was used as an indicator of exposure to HAP and as a proxy for PM exposures (Naeher et al. 2001; Northcross et al. 2010). Passive-diffusion colorimetric CO tubes (Gastec Corp., Kanagawa, Japan) were used, with high-range tubes (range, 1.04–2,000 ppm-hr) for baseline period measurements before stove construction, and low-range tubes (range, 0.4–400 ppm-hr) for observation period measurements. Methods for deploying, validating, and analyzing the CO tubes have been described previously (Smith et al. 2010).

Pregnant women wore CO tubes for a 48-hr period, with a total of 378 CO tubes worn during 247 (of the 254) pregnancies. Baseline measurements were made before stove construction when all houses were using open fires. During this period, women wore 278 CO tubes during their pregnancy, with 4% of women in the first, 33% in the second, and 63% in the third trimester. During the observation period, CO measurements were made at 3- to 6-month intervals. Pregnant women wore 100 CO tubes during this period, with 6% of women in the first, 14% in the second, and 80% in the third trimesters. If a woman had more than one measurement taken during either the baseline or the observation period, an average of the measures was provided for that period. However, with so few measures available for pregnant women in the first two trimesters with the stove in use, we did not have enough information to incorporate these data into the multivariate birth weight models. Instead, the independent variable is actual stove type used during pregnancy.

*Statistical methods.* We used Stata version 11 (StataCorp LP, College Station, TX, USA) for data analyses. Because CO tube data had a right-skewed distribution, both arithmetic and geometric means and respective standard deviations are reported and log-transformed data were used to test for differences between groups. We compared stove groups and birth weight outcomes with Student’s *t*-test for continuous variables, and chi-square tests of significance for categorical variables. We constructed multivariate linear models of birth weight as a continuous outcome among exposed (open fire) and unexposed (stove) women, based on the stove type that was used during the pregnancy. Covariates were included in the multivariate linear regression if *a*) there was evidence of a relationship with either outcome (birth weight) or HAP exposure (stove group, defined as actual stove in use during pregnancy) in the literature, or *b*) there was an observed statistical association (*p* < 0.10) with either outcome or exposure using appropriate parametric or nonparametric hypothesis tests. To investigate potential seasonal effects of HAP on birth weight (e.g., cold weather may lead to more time spent indoors), we examined season of birth in the multivariate model. The three seasons in this region of Guatemala are cold season (15 November to 14 February), warm, dry season (15 February to 30 April), and warm, rainy season (1 May to 14 November). We also examined the effect of season during the three trimesters of fetal growth; interaction terms for season × trimester of stove introduction were included in the regression models. Variables in the model were assessed for multicollinearity using a variance inflation factor (VIF) and were removed if VIF > 10. Outliers were classified using studentized residuals ± 3.0, and a model without outliers was compared with a model that included the “outlier” observations. Leverage plots and partial regression plots were examined to assess any influence individual observations might have on model coefficients. Predicted residuals were plotted against a normal distribution and against the fitted values, to assess for functional form and constant variance.

We used the likelihood-ratio test statistic to compare restricted versus full (saturated) models to determine which model best described the data. We clustered multiple observations on the same woman (e.g., a woman who gave birth to two children during the study period) to derive standard error estimates that would be robust to nonindependent observations. In the final multivariate model, two outlier observations (one each from chimney-stove and open-fire groups) were excluded because they were found to sufficiently influence the homogeneity of the residuals of the data. An adjusted odds ratio (OR) for presence of LBW among open-fire users, compared with chimney-stove users (reference group), was calculated using logistic regression, and CIs were estimated using robust error estimates. *p*-Values < 0.05 were considered significant.

*Ethical approval.* The study received ethical approval from the institutional review boards at the University of California–Berkeley, University of Liverpool, and Universidad del Valle in Guatemala. Trained fieldworkers obtained verbal informed consent from all participating households before the study.

## Results

Because most women in this region do not seek health care until late in pregnancy, 86 (33.8%) women were in their second trimester and 157 (61.8%) were in their third trimester at time of enrollment. Among all women enrolled during pregnancy, the mothers and fathers in the stove group were 2 years younger on average than the open-fire group (Table 1). Women in the chimney-stove group reported fewer pregnancies and live births than did women in the open-fire group. Women whose infants were weighed < 48 hr after birth were less likely to be primiparous than were women whose infants were weighed between 48 hr and 1 week.

**Table 1:** Demographic and clinical characteristics of pregnant women who delivered singleton infants, by randomized stove type and by time of birth weight measurement.

Stove type		Time of birth weight measurement	
Characteristics at baseline examination	Chimney (*n* = 134)	Open fire (*n* = 120)	Within 48 hr (*n* = 174)	< 1 week but > 48 hr (*n* = 34)
Maternal age, years (mean ± SD)*		25.5 ± 6.7	27.4 ± 6.3		26.7 ± 6.7	26.6 ± 7.4
Paternal age, years (mean ± SD; *n* = 251)*		27.6 ± 7.1	30.0 ± 6.9		28.9 ± 7.2	28.9 ± 7.4
Maternal education, years (mean ± SD; *n* = 248)		2.4 ± 2.2	2.1 ± 2.2		2.3 ± 2.3	2.5 ± 2.5
Paternal education, years (mean ± SD; *n* = 232)		4.7 ± 3.2	4.0 ± 3.1		4.3 ± 3.2	4.7 ± 3.2
Altitude, m (mean ± SD)		2,640 ± 175	2,597 ± 188		2,618 ± 183.9	2,658 ± 146.3
Stove in same room as bedroom [*n* (%)]		24 (17.9)	16 (13.3)		28 (16)	2 (5.8)
Eave spaces in kitchen [*n* (%)]						
Completely closed		25 (18.7)	16 (13.3)		25 (14.3)	4 (11.8)
Partially open		57 (41.8)	42 (35.0)		66 (37.9)	17 (50.0)
Completely open		56 (39.5)	62 (51.7)		83 (47.7)	13 (38.2)
Smoker in home [*n* (%)]		28 (20.9)	35 (29.2)		40 (22.9)	11 (32.3)
Traditional steam bath (*temazcal*) [*n* (%)]		114 (85.1)	104 (86.7)		154 (88.5)	29 (85.3)
Dirt floor in main house [*n* (%)]		120 (89.6)	112 (93.3)		160 (91.9)	31 (91.2)
Has electricity in main house [*n* (%)]		98 (73.1)	92 (76.7)		122 (70.1)	22 (64.7)
Economic support (mean ± SD)a,*		4.0 ± 2.2	4.5 ± 2.2		4.5 ± 2.3	4.1 ± 2.2
Crowding (mean ± SD)*b*		7.1 ± 3.0	7.5 ± 2.7		7.1 ± 2.8	7.3 ± 2.7
Total assets (mean ± SD)*c*		1.4 ± 0.9	1.2 ± 0.9		1.3 ± 0.9	1.1 ± 0.6
Weeks pregnant, based on fundal height (mean ± SD)*d*		30.3 ± 6.5	30.8 ± 6.6		29.2 ± 6.8	31.0 ± 6.1
First pregnancy [*n* (%)]		14 (10.5)	12 (10.0)		18 (10.3)	4 (11.7)
Child spacing [*n* (%)]e,**		48 (35.8)	46 (38.3)		4 (2.3)	7 (20.6)
Gravidity (mean ± SD)		4.6 ± 3.0	5.3 ± 2.8		5.2 ± 3.0	5.0 ± 3.2
Live births (mean ± SD)*		2.9 ± 2.3	3.5 ± 2.3		3.4 ± 2.3	3.2 ± 2.6
Maternal height, cm (mean ± SD)		143.7 ± 4.6	144.7 ± 4.6		143.5 ± 4.3	143.6 ± 4.9
Body mass index, kg/m^2^ (mean ± SD; *n* = 239)		23.4 ± 2.4	23.6 ± 2.5		23.6 ± 2.5	22.8 ± 1.8
Blood pressure, mmHg (mean ± SD; *n* = 246)						
Systolic		107.7 ± 8.2	109.4 ± 10.2		108.1 ± 9.1	108.9 ± 8.7
Diastolic		66.8 ± 7.8	68.3 ± 8.2		67.2 ± 8.3	67.6 ± 8.3
Season of birth [*n* (%)]						
Cold and dry		45 (33.6)	39 (32.5)		27 (15.5)	22 (62.9)
Warm and dry		12 (9.0)	11 (9.2)		17 (9.8)	4 (11.4)
Warm and rainy		77 (47.4)	70 (58.3)		130 (74.7)	9 (25.7)
**a**Ratio of household dependents to household workers. **b**Ratio of people to number of rooms. **c**Asset index is a 0–6 item measure of reported ownership of radio, television, refrigerator, bicycle, motorcycle, or car/truck. **d**Fundal height measured by physician at first prenatal physical examination. **e**Has older sibling ≤ 24 months of age. *Difference between chimney-stove and open-fire groups for maternal age (*t*-test = 2.37; *p* = 0.02), paternal age (*t*-test = 2.78; *p* = 0.005), economic support (*t*-test = 1.98; *p* = 0.04), and live births (*t*-test = 2.07; *p* = 0.04). **Difference between infants weighed at < 48 hr and at 48 hr to 1 week for child spacing (χ^2^ = 18.9; *p* < 0.001).						

Additionally, we found no statistically significant differences in the number of miscarriages (10.8% chimney stove vs. 10.3% open fire, *p* = 0.72). Seventeen women (7%) self-reported a personal history of anemia, with no significant difference between groups (*p* = 0.58). Mean systolic and diastolic blood pressures (mean ± SD) were 1–2 mmHg higher in the open-fire (systolic, 109.4 ± 10.2 mmHg; diastolic, 68.3 ± 8.2 mmHg) than in the chimney-stove group (systolic, 107.7 ± 8.2 mmHg; diastolic, 66.8 ± 7.8 mmHg), but these differences were not statistically significant. Only one woman had borderline hypertension on examination. None of the women had glycosuria, and < 3% had proteinuria or white blood cells (renal disease) based on initial urine exam, with no difference between the two groups (*p* = 0.65).

Characteristics that were associated with use of an open fire versus a chimney stove during pregnancy (*p* ≤ 0.10) among the 174 births in this analysis were less economic support (*p* = 0.10), older maternal age (*p* = 0.07), higher fundal height at baseline pregnancy exam (*p* = 0.001), and higher systolic and diastolic blood pressure at baseline medical exam (*p* = 0.09 and *p* = 0.01, respectively). Variables related to LBW among the 174 births (categorical outcome), at the *p* ≤ 0.10 level, were less economic support (*p* = 0.09), lower maternal height (*p* = 0.10), increased live births (*p* = 0.09), lower fundal height (*p* = 0.007), and birth during the rainy, warm season (*p* = 0.04). Infant sex was unrelated to exposure (*p* = 0.91) and outcome (*p* = 0.45), and inclusion of this covariate did not improve model fit. Although economic support was related to both the exposure and the outcome, it was not included in the final multivariate model because it did not improve model fit.

We measured CO exposures in 247 pregnancies during the baseline and observation periods (Table 2). Because women may cook more (or less) during each trimester, we assessed differences by trimester and by stove type used during pregnancy. At baseline, when all women still used an open fire, we found no statistically significant differences in CO measurements between pregnant women who subsequently used a chimney stove during pregnancy and those who did not. During the observation period, CO exposures differed significantly between the two stove groups during the second and third trimester, and we combined them for all trimesters. Overall, CO exposure was 39% lower in the stove group than in the open-fire group, with mean (± SD) 48-hr values of 4.1 ± 3.2 ppm and 2.5 ± 2.5 ppm, respectively (*p* = 0.0003).

**Table 2:** Personal 48-hr CO exposures (*n* = 378, ppm) during 247 pregnancies, by stove type used during pregnancy and trimester, in baseline and observation periods.

First trimester			Second trimester			Third trimester			Total		
Study period	Open fire	Chimney	Open fire	Chimney	Open fire	Chimney	Open fire	Chimney
Baseline, all still using open fires*a*																
Measures*b*		7		4		47		46		124		50		178		100
Mean ± SD		2.5 ± 1.0		2.7 ± 1.1		3.0 ± 3.2		3.3 ± 3.7		2.8 ± 2.5		2.7 ± 2.3		2.8 ± 2.7		2.9 ± 3.0
GM (GSD)		2.3 (1.5)		2.4 (1.6)		2.2 (2.1)		2.3 (2.1)		2.2 (1.8)		2.1 (2.1)		2.2 (1.9)		2.2 (2.1)
Range		1.2–4.2		1.2–3.6		0.5–19.8		0.6–20.3		0.5–21.7		0.4–12.8		0.5, 21.7		0.4, 20.3
During observation period*c*																
Measures*b*		0		4		6		7		48		35		54		46
Mean ± SD				2.9 ± 1.1		2.9 ± 1.6		1.2 ± 0.7		4.3 ± 3.4		2.7 ± 2.7		4.1 ± 3.2		2.5 ± 2.5
GM (GSD)				2.8 (1.6)		2.4 (2.1)		1.1 (2.2)		3.3 (1.9)		1.9 (2.2)		3.2 (1.9)		1.8 (2.1)
Range				1.5–3.9		0.8–4.9		0.7–2.5		0.7–15.9		0.3–13.2		0.7–15.9		0.3–13.2
Abbreviations: GM, geometric mean; GSD, geometric standard deviation. **a**Using log-transformed CO, no significant differences were detected at baseline between chimney-stove and open-fire groups. **b**Thirty-eight measures were taken on the 11 “second” pregnancies that occurred during the observation period. **c**During the observation period, significant differences in log-transformed CO between stove and open-fire groups during second trimester (*t*-test = 2.28; *p* = 0.04), third trimester (*t*-test = 3.15; *p* = 0.002), and total observations (*t*-test = 3.73; *p* = 0.0003).																

Thirty-nine (22.4%) of the 174 infants measured < 48 hr were LBW (Table 3). Compared with women who used an open fire, the estimated odds of delivering an LBW baby were 26% lower (adjusted OR = 0.74; 95% CI, 0.33–1.66) among women who used a chimney stove during pregnancy, after adjusting for gravidity, maternal height, maternal diastolic blood pressure, and season of birth.

**Table 3:** LBW by stove type [*n* (%)] and estimate of effect of stove type on LBW.

Stove type	Total (*n* = 174)	LBW (*n* = 39)	Normal birth weight (*n* = 135)	Adjusted OR*a*	95% CI
Open fire		105 (100)		26 (24.8)		79 (75.2)		Referent		
Chimney		69 (100)		13 (18.8)		56 (81.2)		0.74		0.33–1.66
**a**Adjusted for maternal height, gravidity, maternal diastolic blood pressure, and season of birth.										

The mean estimated birth weight for the 174 infants was 2,755 g (95% CI, 2,696–2,815 g). Unadjusted linear regression models of the mean gram difference in infant birth weight by stove type used during pregnancy demonstrated that average birth weight was 68 g higher (95% CI, –56 to 191 g) among births to mothers who used the chimney stove versus open fire during pregnancy (Table 4). The estimated difference was greater after adjustment for maternal height, gravidity, maternal blood pressure, and season of birth (89 g; 95% CI, –27 to 204 g).

**Table 4:** Difference in birth weight associated with chimney stove use based on unadjusted and adjusted linear regression models.

Unadjusted model			Adjusted model*a*		
Mean birth weight, g (95% CI)	β-Coefficient, g (95% CI)	*p*-Value	β-Coefficient, g (95% CI)	*p*-Value
Stove type										
Open fire (*n* = 105)		2,729 (2,654 to 2,804)		Referent						
Chimney (*n* = 69)		2,797 (2,697 to 2,896)		68 (–56 to 191)		0.28		89 (–27 to 204)		0.13
Timing of stove introduction										
First/second trimester (*n* = 17)		2,732 (2,526 to 2,937)		Referent						
Third trimester (*n* = 52)		2,796 (2,684 to 2,907)		63 ( –157 to 285)		0.56		117 (–96 to 331)		0.27
**a**Adjusted for maternal height, gravidity, maternal diastolic blood pressure, and season of birth.										

In the final multivariate model, the relationship between stove type used during pregnancy and birth weight was not statistically significant. However, maternal height, parity, and season of birth were statistically significant predictors of birth weight, and maternal diastolic blood pressure approached statistical significance. For every 1-cm increase in maternal height, birth weight increased 17 g (95% CI, 4–30 g) after controlling for stove type, gravidity, maternal diastolic blood pressure, and season of birth. For every past pregnancy, birth weight increased 21 g (95% CI, 2–40 g) after controlling for other variables in the model. If born during the cold season (15 November to 14 February), the infant weighed, on average, 296 g more (95% CI, 109–482 g) than if born during the warm, dry season (15 February to 30 April) or the warm, rainy season (1 May to 14 November), after controlling for other variables in the model. For every 1-mmHg increase in maternal diastolic blood pressure (within the observed range of 40–103 mmHg), infant weight was predicted to increase by 5 g (95% CI, –2 to 12 g) after controlling for stove type, gravidity, maternal height, and season of birth. Sixteen percent of the variance in birth weight was explained by the predictor variables, and the stove explained only 2% of this variance, when we held all of the other variables constant. Although we made all attempts to reduce the root mean square error in modeling the birth weight outcome, the best reduction achievable was 355 g, which explains the wide 95% CIs around the estimates.

Trimester when the stove was introduced was not significantly associated with percentage LBW or gram difference in birth weight between the two groups. Compared with infants born to mothers who used open fires during the entire pregnancy (*n* = 105), infants born to mothers who used the stove during the first and second trimesters weighed 328 g more (95% CI, –180 to 836 g), and infants born to mothers who started using the stove in the third trimester weighed 106 g more (95% CI, –19 to 232 g), after controlling for maternal height, gravidity, maternal diastolic blood pressure, and season. In the subgroup of 69 women who had a chimney stove in use during pregnancy, 52 (75%) had a stove ready for use in the third trimester (mean = 45 days; range, 1–90 days), 15 (22%) in the second trimester (mean = 127 days; range, 93–165 days), and 2 (3%) for the entire pregnancy. Among the women who had the chimney stove in the first or second trimester, five (29.4%) had LBW infants; among women who had the chimney stove in the third trimester, eight (15.4%) had LBW infants.

We also evaluated timing of exposure by trimester of pregnancy using a trimester × cold season interaction term, but the small sample size restricted our ability to detect an interaction effect. Because season and blood pressure may be either collinear with the exposure or on the causal pathway, we examined the models without controlling for these covariates and found similar results.

## Discussion

We estimated a potentially clinically significant (although not statistically significant) 89-g increase (95% CI, –27 to 204 g) in birth weight among infants born to mothers using wood-fueled chimney stoves compared with those born to mothers using open fires. These results are similar to those of four other studies that assessed the relationship between birth weight and maternal exposures to HAP (Boy et al. 2002; Mishra et al. 2004; Siddiqui et al. 2008; Tielsch et al. 2009). Unlike previous studies, however, ours introduced a standardized, improved chimney stove, assessed stove conditions on a weekly basis, and measured personal CO exposures among pregnant women every 3–6 months. In a recent meta-analysis of five studies, which included the present findings, Pope et al. (2010) estimated a reduced mean birth weight of 95.6 g (95% CI, 68.5–124.7 g) and a 38% increase in LBW among women exposed to HAP compared with women who used cleaner stoves (improved wood-fired chimney stoves, gas or electric stoves) during pregnancy (pooled OR = 1.38; 95% CI, 1.25–1.52).

Previous studies compared only birth weight outcomes and maternal report of fuel type. A Zimbabwean study of 3,559 newborns showed that infants born to clean fuel users (liquefied petroleum gas, natural gas, or electricity) weighed 175 g more (95% CI, 50–300 g) than did infants born to mothers who used wood, straw, and dung as fuel (Mishra et al. 2004). In a study of 1,771 newborns in Guatemala, Boy et al. (2002) reported that infants born to women using cleaner-fuel stoves versus wood-fuel stoves weighed 63 g (*p* = 0.05) more, on average, whereas infants born to women using wood-fueled improved stoves were 32 g heavier, on average, than were those born to women using open fires. Some of the improved stoves in that study were poorly maintained or repaired, which may have biased the estimate toward the null. A study of 634 infants in Pakistan found a nonsignificant decrease in birth weight in infants born to women using wood fuel compared with infants born to natural gas users (–82 g; 95% CI, –170 to 9 g) (Siddiqui et al. 2008). Although this last estimate is similar to that observed in the RESPIRE trial, all the study women from RESPIRE used wood-fueled stoves. At present, in these rural, poor communities of Guatemala, an improved wood stove is the only viable, low-cost option.

Despite limitations of a small sample size, possible birth weight measurement error due to delay between time of birth and time of measurement, exposure misclassification, other unmeasured exposures to wood smoke (e.g., visits to other homes with open fires), and air pollution reductions primarily restricted to the third trimester, we identified a potentially clinically significant, but not statistically significant, difference in birth weight. Because of possible exposure misclassification among women who received their stove days before birth, we created an exposure variable indicating whether women had a stove in use for at least 60 days before delivery. This reduced the stove group from 69 to 34 births, introduced uncertainty into the model, and thus reduced our power to detect an effect from stove type with an estimated 63 g increase (95% CI, –103 to 230 g). Because of the heavy demands on field personnel to meet the primary aims of the RESPIRE trial, we were not able to assess newborn gestational age at the home deliveries and are thus not able to state what proportion of LBW was due to preterm birth. In 2009, the primary author trained 10 local traditional birth attendants who work in the communities that previously participated in RESPIRE. Using a cell-phone notification system, traditional birth attendants notified us of home deliveries. Within a 2-week period, we evaluated within 48 hr of birth 22 live newborns who were delivered at home. Preliminary analysis of estimated mean gestational age assessment using the New Ballard scale (Ballard et al. 1991) was 36.1 weeks (SD = 1.4) (Thompson LM, Levi AJ, Bly KC, Ha C, Keirns T, Romero C, unpublished observations). We estimated the lowest gestational age at 32 weeks, with an instrument margin of error of ± 2 weeks. We plan to conduct future studies to extend training in gestational age assessment using validated methods such as the New Ballard scale.

A further limitation of the present study was the small number of maternal CO exposure measurements. A recent systematic review of 41 studies in industrialized countries examined the effect of a range of ambient air pollutants on adverse birth outcomes; 13 studies looked at CO and LBW (Shah et al. 2011). Although averaging times and exposure during pregnancy intervals varied across the studies, significant estimated effects on birth weight were reported for CO as low as 1–2 ppm (of continuous exposure) in the third trimester based on in single-pollutant ambient models (Maisonet et al. 2001). These levels were lower than the 48-hr personal CO concentrations among the pregnant women in RESPIRE, including the stove group.

Another limitation is the lack of information on diet and maternal weight gain during pregnancy. Chronic undernutrition was marked among the participating mothers and is reflected in their short stature. In this study, 2.0% of nonpregnant women were below the standard reference cutoff of 18.5 kg/m^2^ for maternal undernutrition. This is comparable with the 2002 Guatemalan maternal–child health national survey, which found maternal undernutrition to be 1.9% for women of reproductive age (PAHO 2007).

Our study has several strengths. This study of HAP exposure and birth weight is the first to explore a trimester effect with the use of improved stoves, although few women were observed starting from the first or the second trimester. Differential exposure was limited primarily to the third trimester and after stove construction. The velocity of fetal growth is largest during the third trimester, so limiting smoke exposures during this period is crucial (Lieberman et al. 1994). We observed a nonsignificant mean increase of 106 g (95% CI, –19 to 232 g) in infant birth weight among the 52 women who used the chimney stove in the third trimester compared with women who used open fires throughout their pregnancy. Dejmek et al. (2002) estimated that newborns whose mothers were exposed to ETS throughout their pregnancy weighed 53 g less (95% CI, –24 to –82 g), on average, than those whose mothers were unexposed to ETS. In the same study, women who were moderate cigarette smokers (1–10 cigarettes/day) during the third trimester had children whose birth weight averaged 130 g less (95% CI, 95–176 g) than that of children born to mothers who never smoked. Our estimate falls within a plausible range for this third-trimester exposure period.

A further strength of our study is the low prevalence of active tobacco smoke and ETS exposure. In other studies measuring the impact of solid fuels used for cooking, LBW could potentially be attributed in part to unmeasured tobacco exposures if smoking is common. In Pakistan, Siddiqui et al. (2008) found that 15% of the study women smoked during pregnancy. Among women enrolled in RESPIRE, only one woman stated that she was a former smoker, and among the 26% of women who reported a smoker in the house, only one or two cigarettes were smoked per day.

Weekly visits made by trained fieldworkers ensured that the chimney stove was functioning as intended. During the RESPIRE trial, the chimney stove reduced kitchen air pollution by approximately 90%. Among all 529 women (pregnant and not pregnant) monitored during the 2-year trial, arithmetic mean personal exposures over 48 hr were reduced by 54%, from 4.8 ± 3.6 ppm to 2.2 ± 2.6 ppm, in the chimney-stove group (Smith et al. 2010). Among pregnant women, CO was 39% lower among women who used the cook stove compared with open-fire users. However, CO levels were high even among chimney-stove users.

In these communities, there are other important sources of smoke exposure, and certainly there are exposures to other copollutants in wood smoke besides CO. Roughly 85% of the population uses a wood-fired steam bath for 30–45 min several times a week (Spanish *temazcal*, Mam *chuj*). *Temazcal* use could have a much greater effect on LBW than kitchen exposures. Data presented here did not incorporate these significantly elevated exposures, because women were asked to remove their CO tubes before entering the *temazcal* to bathe, because high-humidity conditions would interfere with the accuracy of the tube measurement. These extreme, acute CO exposure levels affect the entire population of *temazcal* users and could contribute to the high incidence of LBW observed in the RESPIRE population (Thompson et al. 2011). In this study, however, women who had the chimney stove in use during pregnancy and those who used the open fire were equally exposed to the *temazcal* (86.9% vs. 89.5%; *p* = 0.60).

We observed a strong association with season, with a significantly higher average birth weight among children born during the cold season compared with children born during the rest of the year. We anticipated potentially higher exposures to CO during the cold season, when household members typically sit around the fire for warmth. However, we found no seasonal differences in measured maternal CO exposures (data not shown). It is possible that higher birth weight during the cold season is attributable to increased food availability during the harvest period, which occurs in the months preceding the cold season. Increased fetal weight may be attributable to improved nutrition during the second trimester and third trimester, when fetal weight gain accelerates, although this cannot be verified from our data. Rao et al. (2009) found evidence of a seasonal energy stress effect among Indian women in rural farming communities. Women who had higher maternal caloric intake (based on 24-hr recall method) during the second and third trimesters, which coincided with the winter harvest season, were found to have heavier newborns than did women whose second and third trimesters coincided with summer and monsoon seasons (Rao et al. 2009). Women in the RESPIRE trial not only face chronic malnutrition (as demonstrated by their short stature and low body mass index) but also experience seasonal stress that acutely affects infant birth weight.

A final strength of this study is the intensive, weekly household surveillance that was employed during RESPIRE. This increased our ability to weigh infants born at home soon after birth. Our LBW prevalence was 22.4%, almost double the national reported prevalence of 12% (PAHO 2007). The national LBW rate includes urban populations, typically characterized by higher socioeconomic status, better access to medical care, and cleaner cooking fuels.

## Conclusion

To the best of our knowledge, this is the first study to monitor stove use on a weekly basis, measure CO exposures during pregnancy, and explore the impact of wood smoke, by trimester of exposure, on birth weight. Although we randomized pregnant women to a stove intervention during RESPIRE, most of the women were in their third trimester; given the timing of stove allocation, an intention-to-treat analysis was not possible. Although the chimney stove reduced exposure to HAP, exposures remained high for many women and infants. Exposure reduction through well-operating chimney stoves would not only improve the quality of life of the cook and her family but also reduce LBW prevalence, based on evidence from ambient and HAP studies. Future research is needed to quantify women’s personal exposure to HAP during each trimester of pregnancy to assess the effect on adverse newborn outcomes, including birth weight and preterm birth. This would provide supportive evidence to encourage delivery of improved stoves and other clean energy technologies to millions of women and their families in low-income countries who continue to burn solid fuels for cooking and heating.
